# Back-Gate GaN Nanowire-Based FET Device for Enhancing Gas Selectivity at Room Temperature

**DOI:** 10.3390/s21020624

**Published:** 2021-01-17

**Authors:** Md Ashfaque Hossain Khan, Ratan Debnath, Abhishek Motayed, Mulpuri V. Rao

**Affiliations:** 1 Department of Electrical and Computer Engineering, George Mason University, Fairfax, VA 22030, USA; 2 N5 Sensors, Inc., Rockville, MD 20850, USA; rdebnath@n5sensors.com (R.D.); amotayed@n5sensors.com (A.M.)

**Keywords:** gas sensor, cross-sensitivity, gallium nitride (GaN), metal oxide, back-gate FET

## Abstract

In this work, a TiO_2_-coated GaN nanowire-based back-gate field-effect transistor (FET) device was designed and implemented to address the well-known cross-sensitive nature of metal oxides. Even though a two-terminal TiO_2_/GaN chemiresistor is highly sensitive to NO_2_, it suffers from lack of selectivity toward NO_2_ and SO_2_. Here, a Si back gate with C-AlGaN as the gate dielectric was demonstrated as a tunable parameter, which enhances discrimination of these cross-sensitive gases at room temperature (20 °C). Compared to no bias, a back-gate bias resulted in a significant 60% increase in NO_2_ response, whereas the increase was an insignificant 10% in SO_2_ response. The differential change in gas response was explained with the help of a band diagram, derived from the energetics of molecular models based on density functional theory (DFT). The device geometries in this work are not optimized and are intended only for proving the concept.

## 1. Introduction

For a gas sensor to be widely accepted for Internet of Things (IoT) applications, it must have high response, precise selectivity, quick response–recovery, low cost, small size, low power, long operating life, and stable operation across various environmental conditions. Unfortunately, no such gas sensor has yet been reported having all these desired properties. Metal oxide-based sensors have been employed to detect toxic environmental gases for several years [1]. They have the capability of providing all the above-mentioned gas sensing properties except precise selectivity. This is because their sensing mechanism generally involves the chemical interaction between the gas molecule and the oxygen chemisorbed on the sensing surface [2]. Gas molecules having similar chemical properties take part in this oxygen interaction, irrespective of the oxidizing or reducing nature of metal oxides [3]. Thus, cross-sensitivity among different analyte gases is inevitable in a metal oxide-based sensor device. For instance, the detection of NO_2_ and SO_2_ gas is hampered due to cross-interference when emitted in a mixed condition from a stationary source [4]. Systematic variations in the parameters such as dopants, additives, operating temperatures, bias voltage, grain size, and morphology have been adopted to achieve the necessary selectivity among various analytes [5]. Although efforts have been made, the problem of cross-sensitivity cannot yet be fully eliminated. This oxide-inherent cross-sensitive issue of a chemiresistive sensor can be resolved by employing several techniques, including a sensor array [6,7] and a field-effect transistor (FET) sensor [8].

Nanowires exhibit one-dimensional nanostructures that offer large surface-to-volume ratio, suitable for gas-sensing applications [9,10]. In the past few years, several nanowire-based gas sensors have been reported showing enhanced sensing performance in comparison to their bulk counterpart [11,12,13]. However, the analyte selectivity issue remains a challenge to be resolved, especially for metal-oxide sensors [14,15]. In this work, back-gate configuration was exploited in a GaN nanowire FET sensor for the differentiation of cross-sensitive gases. A Si back gate with C-AlGaN as the gate dielectric was formed on a TiO_2_/GaN nanowire to develop the back-gate FET device. Electrical and gas characterizations were conducted on the sensor devices in the presence of UV light at room temperature. Then, gas response enhancement and the sensing mechanism were described using an energy band diagram based on density functional theory (DFT) molecular models.

## 2. Experimental Details

**Figure 1:**
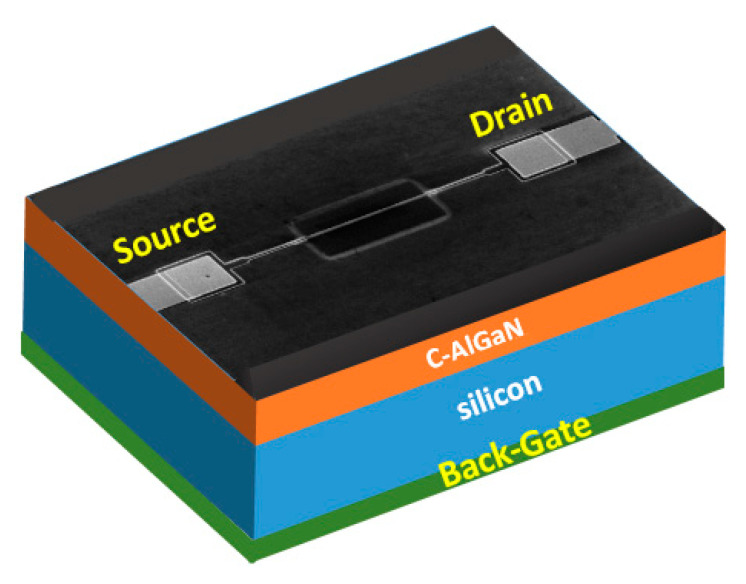
Fabricated TiO_2_/GaN nanowire field-effect transistor (FET) sensor. FESEM image of the two terminals. The TiO_2_/GaN device is shown on the top side.

Here, GaN nanowires were made from the Si-doped GaN epilayer using the top-down fabrication process. They were developed on a silicon substrate with a combination of industry standard stepper lithography and inductively coupled plasma (ICP) etching of a GaN/AlGaN epilayer grown on a Si substrate. The nanowire size was observed as being quite uniform. Having a length and a width of ~10 µm and ~400 nm, respectively. Subsequently, ohmic metal contacts composed of Ti (40 nm)/Al (80 nm)/Ti (40 nm)/Au (40 nm) were deposited upon the nanowire. Next, a passivation layer of SiO_2_ was formed on the nanowire device using the standard plasma-enhanced chemical vapor deposition (PECVD) technique. Later, a functionalization window on the GaN nanowire was developed by reactive-ion etching (RIE) of the SiO_2_ layer. A thin layer (5–10 nm) of TiO_2_ nanoclusters was deposited on the nanowire surface by RF magnetron sputtering followed by rapid thermal annealing (RTA). The fabrication details and process flow diagram of the nanowire-based two-terminal device can be found in our previous papers [16,17,18,19]. The C-doped AlGaN buffer layer (~200 nm) formed in between the GaN epilayer and the Si substrate was used as the gate dielectric here to develop a back-gate FET configuration (Figure 1). This dielectric layer was grown on top of the 300 µm thick Si substrate. To best of our knowledge, this is the first back-gate bias study using AlGaN as a gate dielectric in GaN-nanostructured devices. Finally, the fabricated FET device was placed and wire-bonded to a 24-pin ceramic dual in-line package (DIP).

All the current–voltage measurements of the FET device were performed using a National Instrument (NI) PXI SMU system (Rockville, MD, USA) under an LED UV light source having a wavelength of 365 nm and a power of 470 mW/cm^2^. The FET sensor was inserted in a mini gas chamber made of stainless steel for obtaining gas responses. Then, a mixture of NO_2_ or SO_2_ gas and breathing air was introduced into the chamber with a net flow (air + gas) of 0.5 slpm. The device current response was collected by the NI PXI SMU system and converted to a resistance value. Sensor response was evaluated as (Rg-Ra)/Ra, where Rg and Ra are resistances in the presence of the gas–air mixture and air, respectively.

## 3. Results and Discussion

The device properties of the fabricated GaN nanowires were discussed in detail in our previous work [20]. Here, the nanowire depletion region thickness and, consequently, its resistance was modulated by applying the back-gate bias voltage. Due to the thick dielectric layer and substrate used in this study, the fabricated FET requires a relatively high back-gate voltage, but it can be reduced by optimizing the thicknesses of the AlGaN layer and the Si substrate.

**Figure 2:**
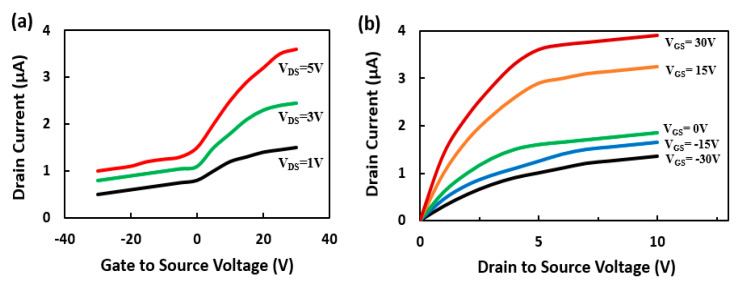
(**a**) *I*_DS_ vs. *V*_GS_ plot for the GaN nanowire-based FET device for a *V*_DS_ of 1, 3, and 5 V. (**b**) I_DS_ vs. *V*_DS_ plot for the GaN nanowire-based FET device with *V*_GS_ varied from −30 V to 30 V at a step of 15 V. All the measurements were done under UV light at room temperature (20 °C).

Since Si-doped GaN nanowire exhibits n-type behavior, the developed nanowire-based FET showed n-channel field-effect transistor characteristics operating in the depletion mode. When a bias voltage is applied to a Si back gate, the drain-to-source current is modulated within the GaN nanowire. Figure 2a demonstrates the drain current behavior with respect to V_GS_ varied from −30 V to +30 V at a step of 5 V. The drain-to-source voltage (V_DS_) was kept constant at 1, 3, and 5 V. Figure 2b shows the plot of I_DS_ vs. V_DS_ for the GaN nanowire FET device with V_DS_ varied from 0 V to 10 V. The drain currents presented here are for back-gate (V_GS_) voltages of −30 V, −15 V, 0 V, 15 V, and 30 V. The FET shows a typical drain current saturation with knee voltage at about 5 V. The electron field effect mobility (µ) was calculated using the following equation: [21,22]

(1) $$\mu =\frac{g_{m}L\ln\left(4t_{\mathrm{ox}}/d\right)}{V_{DS}2{\Pi \varepsilon }_{o}\varepsilon }$$

where the transconductance, *g_m_*, is the slope of the *I*_DS_/*V*_GS_ plot for a particular V_DS_, *L* and *d* are the length and width of the nanowire, respectively, t_ox_ is the gate dielectric thickness, and ε is the series permittivity of Si and AlGaN. Maximum *g_m_* derived from the plot was 0.07 µA/V at a V_DS_ of 5 V, and the corresponding mobility was calculated as 112 cm^2^ V^−1^ s^−1^, which is close to the Hall measurement value of 105 cm^2^ V^−1^ s^−1^.

**Figure 3:**
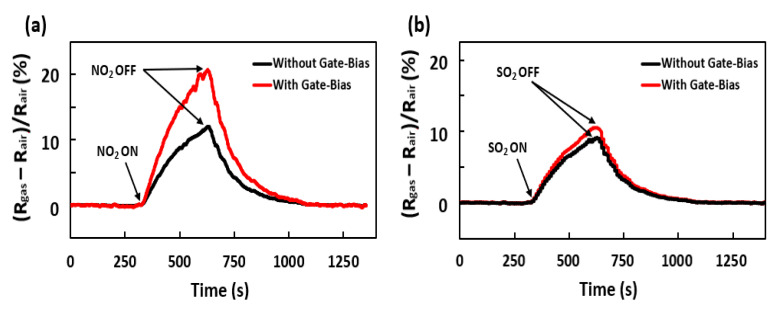
Sensor response to 10 ppm of (**a**) NO_2_ and (**b**) SO_2_ with and without applying gate bias voltage of 30 V under UV light in dry air at room temperature (20 °C).

The gas sensing data were obtained in dry air under UV light at room temperature. The device was allowed to obtain a stable baseline signal by flowing dry air for 10 min before exposing it to the analyte gas for 250 s. When the gas flow was turned off, the sensor was kept for 10 min for baseline recovery without any purging. The normalized resistance responses of the TiO_2_/GaN nanowire-based sensor device when exposed to 10 ppm of NO_2_ and SO_2_ gas are shown in Figure 3. Without any gate bias, the sensor acts as a two-terminal resistor and exhibits a similar response magnitude for the two target gases. It is clearly seen that, upon applying a positive gate bias of 30 V, the NO_2_ response was enhanced by almost a 60% increase, whereas the SO_2_ response remains almost the same with only a marginal 10% increase. Thus, the selectivity of the FET sensor was improved for NO_2_ gas due to the lowering of cross-sensitive interference from SO_2_ gas. The change in sensor responses is mainly attributed to the modification of the channel depletion region with gate bias voltage. Consequently, the device’s Fermi energy level is shifted and the charge transfer between gas and sensor surface is altered. We fabricated a total of five FET devices of the same type and performed similar electrical and gas characterizations. It was observed that all of the back-gate FET sensors exhibited similar differential enhancement of responses toward the two analyte gases, indicating an insignificant device variability.

## 4. Gas-Sensing Mechanism

The gas-sensing mechanism of the TiO_2_/GaN nanowire-based device was explained with the help of an energy band diagram and a charge transfer process between the metal oxide and the gas molecule. The charge transfer process between the metal oxide surface and gas molecules controls the level of chemical interaction between them. It is well known that direction and value of charge transfer in the adsorption system depend on the work function of sensing material as well as the highest occupied molecular orbital (HOMO) and lowest unoccupied molecular orbital (LUMO) of the target gas. The charge transfer continues to take place until equilibrium Fermi energy is reached within the adsorption system.

**Figure 4:**
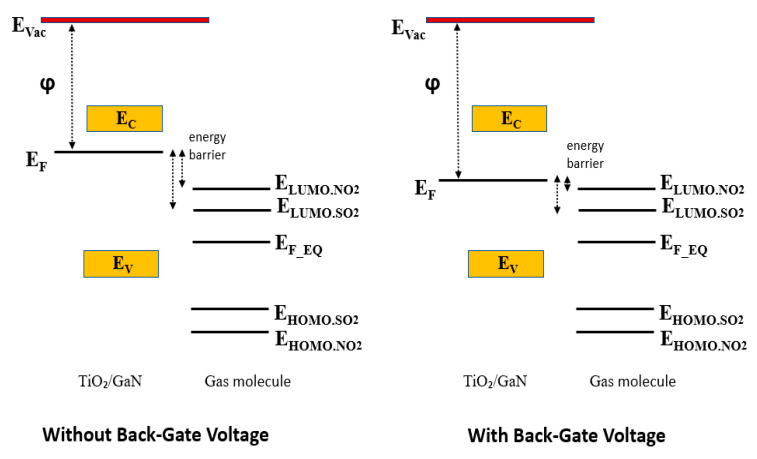
Schematic representation of the energy band diagram showing energy barriers between the TiO_2_/GaN nanocomposite and the gas molecule. Here, ϕ, E_C_, E_V_, and E_F_ represent work function, conduction band, valence band, and Fermi energy of TiO_2_/GaN, respectively, E_Vac_ denotes the energy of vacuum level, and E_F_EQ_ represents the equilibrium state of the adsorption system. E_LUMO_ and E_HOMO_ indicate the orbital energies of the gas molecules. LUMO = lowest unoccupied molecular orbital; HOMO = highest occupied molecular orbital.

A schematic of the energy band diagram showing the energy barrier between the TiO_2_/GaN system and the gas molecule is shown in Figure 4. The Fermi energy of the TiO_2_/GaN system and the HOMO and LUMO of NO_2_ and SO_2_ used here were obtained from our previous study on molecular models of adsorption systems using first-principle calculations within DFT [23,24].

Since the energy gap between the Fermi energy in TiO_2_/GaN and the LUMO is much less than that of the HOMO, electrons from the sensing surface prefer to move toward the LUMO of the gas molecule during adsorption. Here, the electrons are transferred from the metal oxide to the gas molecule by the process of quantum tunneling, which can be described by a single-step barrier concept [25]. It is well known that the probability of charge transmission through the energy step increases exponentially with the decrease in barrier height [26]. After applying the gate bias, the LUMO of NO_2_ was much closer to the TiO_2_/GaN Fermi level than the LUMO of SO_2_. That means that the probability of electron transfer between sensor and gas becomes comparatively higher in the case of NO_2_ adsorption. This increased charge transfer amount, on applying the gate bias, is reflected in the form of a significant gas response change as shown in Figure 3a. Therefore, the TiO_2_/GaN FET sensor becomes strongly selective toward NO_2_ against interfering gases such as SO_2_, with the gate bias being another tunable parameter. The Fermi energy of the two-terminal TiO_2_/GaN device without gate bias voltage, the HOMO and LUMO of NO_2_ and SO_2_ aligned to the vacuum level, as well as the absolute value of energy differences between the LUMO and Fermi energy (E_F-LUMO_) and energy differences between the HOMO and Fermi energy (E_F-HOMO_) are shown in Table 1.

## 5. Conclusions

In this work, we presented a GaN nanowire-based back-gate FET sensor device to address the cross-sensitivity among interfering gases NO_2_ and SO_2_. By applying a back-gate bias voltage to the Si substrate of a two-terminal TiO_2_/GaN sensor, selectivity toward NO_2_ was enhanced. It was found that the NO_2_ response was improved by 60% as compared to an insignificant 10% increase in the SO_2_ response, after applying the back-gate bias. The differential gas response due to the back-gate bias was discussed with the help of an energy band diagram and a charge transfer process derived from the DFT energy calculation of a molecular model.

## Figures and Tables

**Table 1:** Fermi energy, molecular frontier orbital energies, and energy differences. E_F-LUMO_ is the absolute value calculated by E_F_ (device)-E_LUMO_ (gas). E_F-HOMO_ is the absolute value calculated by E_F_ (device)-E_HOMO_ (gas).

Adsorption System	Fermi Energy (eV)	LUMO (eV)	HOMO (eV)	E_F-LUMO_ (eV)	E_F-HOMO_ (eV)
TiO_2_/GaN	−2.137	-	-	-	-
NO_2_	-	−2.890	−8.612	0.753	6.475
SO_2_	-	−3.182	−7.015	1.045	4.878

## Data Availability

Not applicable.
